# Real-Time and Meter-Scale Absolute Distance Measurement by Frequency-Comb-Referenced Multi-Wavelength Interferometry

**DOI:** 10.3390/s18020500

**Published:** 2018-02-07

**Authors:** Guochao Wang, Lilong Tan, Shuhua Yan

**Affiliations:** 1 High-tech Institution of Xi’an, Xi’an 710025, China; 15349227983@189.cn; 2 College of Mechatronic Engineering and Automation, National University of Defense Technology, Changsha 410073, China; yanshuhua996@163.com

**Keywords:** absolute distance measurement, frequency comb, multi-wavelength interferometry, direct synthetic wavelength interferometry, non-ambiguous range

## Abstract

We report on a frequency-comb-referenced absolute interferometer which instantly measures long distance by integrating multi-wavelength interferometry with direct synthetic wavelength interferometry. The reported interferometer utilizes four different wavelengths, simultaneously calibrated to the frequency comb of a femtosecond laser, to implement subwavelength distance measurement, while direct synthetic wavelength interferometry is elaborately introduced by launching a fifth wavelength to extend a non-ambiguous range for meter-scale measurement. A linearity test performed comparatively with a He–Ne laser interferometer shows a residual error of less than 70.8 nm in peak-to-valley over a 3 m distance, and a 10 h distance comparison is demonstrated to gain fractional deviations of ~3 × 10^−8^ versus 3 m distance. Test results reveal that the presented absolute interferometer enables precise, stable, and long-term distance measurements and facilitates absolute positioning applications such as large-scale manufacturing and space missions.

## 1. Introduction

Rapid, precise, and long-range absolute distance measurement (ADM) by optical interferometry is of extraordinary importance for industrial applications, such as large-scale aircraft machining, ultra-precision semiconductor manufacturing, and sub-aperture stitching measurement [1,2]. Traditional laser ranging for distance flatly uses a laser-pulse-based time-of-flight method to measure distance with sub-millimeter resolutions. This method is limited in virtue of its electronic resolution because this leads to a far less precise than optical interferometry [3]. Meanwhile, laser interferometers that are based on a homodyne or heterodyne principle adeptly measure displacement with sub-wavelength precision via phase-counting techniques. However, these interferometers are unable to achieve ADM independently because of a 2π phase-ambiguity in single-wavelength interferometers, which substantially restricts the scope of applications [4]. In order to extend the non-ambiguity range (NAR) of laser interferometers, trial solutions based on ordinary laser sources, such as frequency scanning interferometry, have been studied, but these attempts have failed to achieve satisfactory performance [5,6]. The recent advent of frequency combs has brought tremendous improvements to length metrology in both precision and range, and these combs, due to their superior characteristics in both temporal and spectral domains, have made breakthroughs in the realization of ADM [7,8,9]. On the basis of frequency combs, a series of applicable methods, including synthetic wavelength interferometry (SWI) [10,11], dispersive interferometry [12,13], multi-wavelength interferometry (MWI) [14,15,16], frequency modulated continuous wave interferometry [17], time-of-flight method [18,19,20,21], and dual-comb interferometry [22,23,24], have reportedly achieved high precision ADM at varied ranges.

At the present stage, most frequency-comb-based measuring schemes exploit frequency combs as a direct laser source for achieving ADM. However, they can hardly cover a full distance range of meters with nanometer precision in one-shot measurement. For instance, the time-of-flight method using balanced optical cross-correlation readily measures very long distances, but the optimal precision is sub-micrometer, and a distance that is larger than a certain threshold value is a prerequisite due to a narrow tunable range in repetition rate [21]. Another type of time-of-flight methods explores the extremely precise interval length of the repetition pulse as the length gauge; however, this method only measures certain discrete distances based on pulse coherence through auxiliary scanning [20]. Dual-comb interferometry has the capability of measuring long distances fast and readily, but tiny segments of dead zones exist when the measurement pulse and the reference pulse are temporally close [22]. The SWI method measures distance with the precision of hundreds of micrometers [11], and dispersive interferometry obtains relatively short NAR [12]. Frequency-comb-referenced MWI has been shown to manage ADM and facilitate long distance measurement as precisely as conventional laser interferometers do [14,15,16,25]; the frequency comb serves as a wavelength ruler and enables wavelengths of continuous-wave lasers to be precisely calibrated to time and frequency standards.

We sought to develop frequency-comb-referenced MWI to exploit the potential for instantly measuring meter-scale absolute distances. As a result, meter-scale absolute distance measurement was performed in real time instead of in the traditional manner, where multi-wavelengths are operated in sequence. First, four different wavelengths were simultaneously generated by accurately phase-locking them to the frequency comb for parallel heterodyne detections. Second, another free-running laser was merged to properly implement direct synthetic wavelength interferometry. With such integration, we consequently extended the measurable NAR to a meter level, which is highly emphasized in modern large-scale manufacturing, such as the fabrication of ultra-broad flat panel displays and solar cell devices. For performance evaluation, a linear distance comparison over a range of 3.0 m and a 10 h continuous comparison were made to confirm the measurement capability of this ADM prototype.

## 2. Methods

### 2.1. Principle of Multi-Wavelength Interferometry

**Figure 1:**
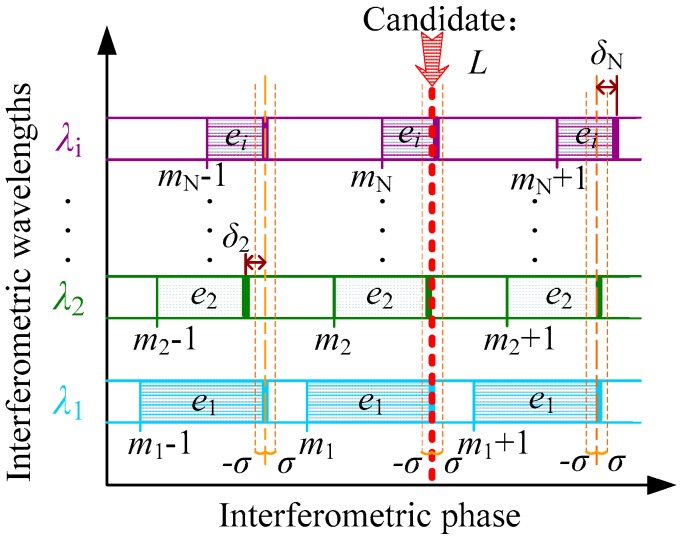
The diagram of the excess fraction method.

In single-wavelength interferometry, the measured length *L* is scaled by the optical wavelength and at sub-wavelength precision, but the periodical phase confines the measurement of NAR to half of the wavelength. Such a plight can be overcome by multi-wavelength interferometry [26]. Given a series of selected optical wavelengths, the target distance *L* is expressed as

(1) $$\left\{ \begin{array}{c} L=\frac{\lambda_{1}}{2n_{1}}\cdot \left(m_{1}+e_{1}\right)\\ L=\frac{\lambda_{2}}{2n_{2}}\cdot \left(m_{2}+e_{2}\right)\\ \cdots \cdots \\ L=\frac{\lambda_{N}}{2n_{N}}\cdot \left(m_{N}+e_{N}\right) \end{array}\right.$$

where $\lambda_{i}$ is the vacuum wavelength, $n_{i}$ is the refractive index of air, $m_{i}$ is a positive integer, $e_{i}$ is the normalized excess fraction for interferometric phase ([0, 1] corresponds to [0, 2π]), and N is the total number of used wavelengths. Apparently, there are N + 1 unknowns involved in Equation (1), namely *L*, *m*_1_, *m*_2_, ⋯ *m*_N−1_, *m*_N_, while the number of equations is only N. Generally, there is no definitely unique solution for Equation (1). If the solution exists, the candidate for *m*_1_ could be a series of arithmetic progression, and the common difference between the two adjacent candidates corresponds to the so-called NAR of MWI. However, allowing for the integer definition of *m_i_* and the reasonable initial guess of *L* (uncertainty is smaller than half of the NAR), *m_i_* is probably identified as a unique value by an MWI iterative algorithm based on the excess fraction method [27]. Figure 1 shows the basic principle of the excess fraction method. When the candidate $m_{i}$ is searched by the excess fraction method in MWI, at first, a variable ${m^{\prime }}_{1}$ within a certain interval is sequentially assumed, and a potential distance value can then be computed as $L^{\prime }=\left({m^{\prime }}_{1}+e_{1}\right)\cdot \lambda_{1}/2$ in the scale of $\lambda_{1}$. Based on that default value $L^{\prime }$, for all other wavelengths, there is a series of phase residues $\delta_{i}$ between the measured phase $e_{i}$ and the nominal fractional phase computed with $L^{\prime }$. These residues $\delta_{i}$ are deduced as

(2) $$\left\{ \begin{array}{l} \delta_{2}=\mathrm{Frac}\left[\frac{2L^{\prime }}{\lambda_{2}/n_{2}}-e_{2}\right]=\mathrm{Frac}\left[\frac{\left(m_{1}+e_{1}\right)\lambda_{1}/n_{1}}{\lambda_{2}/n_{2}}-e_{2}\right]\\ \delta_{3}=\mathrm{Frac}\left[\frac{2L^{\prime }}{\lambda_{3}/n_{3}}-e_{3}\right]=\mathrm{Frac}\left[\frac{\left(m_{1}+e_{1}\right)\lambda_{1}/n_{1}}{\lambda_{3}/n_{3}}-e_{3}\right]\\ \;\;\cdots \cdots \\ \delta_{N}=\mathrm{Frac}\left[\frac{2L^{\prime }}{\lambda_{N}/n_{N}}-e_{N}\right]=\mathrm{Frac}\left[\frac{\left(m_{1}+e_{1}\right)\lambda_{1}/n_{1}}{\lambda_{N}/n_{N}}-e_{N}\right] \end{array}\right.$$

where the function of Frac[*x*] represents extracting the fractional part of the real number *x*. A positive constant of *σ* is denoted as the fractional tolerance, which is usually smaller than the detected precision of phases; then, if all the $\delta_{i}$ converge to an interval of [−σ, σ], the integer of ${m^{\prime }}_{1}$ and the dependent $L^{\prime }$ are identified as the candidates for $m_{i}$ and L. In addition, if there is more than one group of candidates arising, a criterion such as a comparison of maximum ($\left|\delta_{i}\right|$) or $\sum \delta_{i}^{2}$ can be used to screen out false candidates.

### 2.2. Explanation for NAR Extension

The proposed MWI synchronously uses multiple frequency-comb-referenced wavelengths to realize a large NAR in absolute distance measurement. For explicit explanation of the entire scheme for NAR extension, the notion of virtual synthetic wavelength (with the symbol of Λ) is introduced here to show how the chain of NAR extension is formed. First of all, the primary order of the NAR chain, named as NAR_1_, is denoted as half of the smallest wavelength, i.e., $\lambda_{1}/2$. To sequentially introduce the NAR_2_, the first analyzed virtual synthetic wavelength is denoted as $\Lambda_{14}$. It is composed of two wavelengths, $\lambda_{1}$ (the smallest) and $\lambda_{4}$ (the biggest), by the equation below:

(3) $$\frac{1}{\Lambda_{14}}=\left|\frac{1}{\lambda_{1}}-\frac{1}{\lambda_{4}}\right|.$$

Half of $\Lambda_{14}$ offers an extended NAR, named as NAR_2_, and relates to NAR_1_ by the equation below:

(4) $${\mathrm{NAR}}_{2}=\frac{\Lambda_{14}}{2}=\frac{1}{2}\cdot \frac{\lambda_{4}\cdot \lambda_{1}}{\left|\lambda_{1}-\lambda_{4}\right|}=\frac{\lambda_{4}}{\left|\lambda_{1}-\lambda_{4}\right|}\cdot {\mathrm{NAR}}_{1}.$$

According to Equation (4), $\lambda_{4}/\left|\lambda_{1}-\lambda_{4}\right|$ is denoted as the scaling factor $\beta_{2}$ from NAR_1_ to NAR_2_, and then NAR_1_ has been extended to $\beta_{2}\cdot {\mathrm{NAR}}_{1}$. However, to retrieve the integer part of the single wavelength by the excess fraction method, a basic inequality needs to be built involving the precision term (ε) of the virtual synthetic phase. The required format is

(5) $${\mathrm{NAR}}_{2}\cdot \varepsilon <\frac{{\mathrm{NAR}}_{1}}{2}.$$

If the phase measurement precision of single-wavelength interferometry is denoted as σ (fraction), the synthetic phase precision ε can be expressed as $\varepsilon =\sqrt{\sigma_{1}^{2}+\sigma_{4}^{2}}$ ($\sigma_{1}$ and $\sigma_{4}$ are for $\lambda_{1}$ and $\lambda_{4}$, respectively.). Given the condition of parallel and coequal phase detection, ε can be calculated as $\sqrt{2}\sigma$, and Equation (5) can be transformed to

(6) $$\beta_{2}\cdot \sqrt{2}\sigma <\frac{1}{2}.$$

Equation (6) indicates that the scaling factor $\beta_{2}$ and phase precision σ are tightly interrelated. When the case of more wavelengths is considered, the general inequality on the requirement of the scaling factor and phase detection precision for NAR extension is expressed as

(7) $$\beta_{k}\cdot \sqrt{M}\sigma <\frac{1}{2}$$

where $\beta_{k}$ is the scaling factor for the k-th order NAR, and M is the wavelength number used in the newly formed NAR_k_. Equation (7) indicates that the scaling factor decreases with the increment in the wavelength number at the presence of constant phase precision.

**Figure 2:**
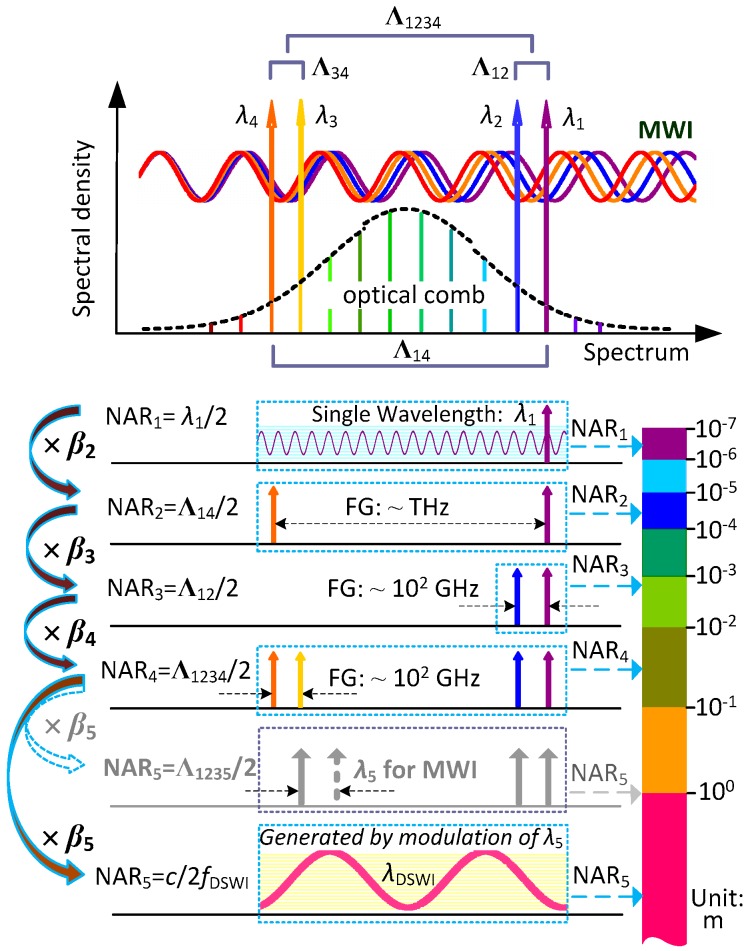
Schematic diagram for non-ambiguity range (NAR) extension. Λ: virtual synthetic wavelength; FG: frequency gap; NAR_i_: i-th order NAR.

From the view of making synthetic wavelength, the aim of the NAR extension is to maximize the length of virtual synthetic wavelength under the basic rule of Equation (7). As depicted in Figure 2, the smallest wavelength $\lambda_{1}$ provides the primary 1st-order NAR_1_ (sub-micrometer level) in the finest subdivision. The far-separated $\lambda_{1}$ and $\lambda_{4}$ are matched to yield a virtual synthetic wavelength of $\Lambda_{14}$, which offers the 2nd-order NAR_2_ (tens of micrometers level) with a scaling factor of $\beta_{2}$. Similarly, the near-separated wavelengths of $\lambda_{1}$ and $\lambda_{2}$ make a longer virtual synthetic wavelength $\Lambda_{12}$, offering the 3rd-order NAR_3_ (several millimeters level) with a scaling factor of $\beta_{3}$. The scaling factor for $\Lambda_{12}$ could be theoretically promoted with a pair of closer wavelengths, but the frequency gap for wavelength de-multiplexing in the synchronous multi-wavelength interferometer ought to be practically considered. The permissible frequency spacing of the FBG channel used for wavelength separation and recombination (C-band) is close to 100 GHz, so NAR_3_ is limited to several millimeters. To circumvent this limitation of wavelength separation and recombination, NAR extension is managed with $\Lambda_{1234}$ formed by a secondary virtual synthesis of $\Lambda_{12}$ and $\Lambda_{34}$. That virtual synthetic wavelength $\Lambda_{1234}$ involves four wavelengths ($\lambda_{1}<\lambda_{2}<\lambda_{3}<\lambda_{4}$) simultaneously and follows the equation

(8) $$\frac{1}{\Lambda_{1234}}=\left|\frac{1}{\Lambda_{12}}-\frac{1}{\Lambda_{34}}\right|=\left|\left|\frac{1}{\lambda_{1}}-\frac{1}{\lambda_{2}}\right|-\left|\frac{1}{\lambda_{3}}-\frac{1}{\lambda_{4}}\right|\right|$$

where $\lambda_{3}$ is the further added wavelength for the formation of NAR_4_ based on the existence of $\lambda_{1}$, $\lambda_{2}$, and $\lambda_{4}$. According to Equation (8), there are four wavelengths used in the synthesis, so the scaling factor $\beta_{4}$ (equal to $\Lambda_{34}/\left|\Lambda_{12}-\Lambda_{34}\right|$) has to be decreased by about $\sqrt{2}$ compared with $\beta_{2}$ and $\beta_{3}$ according to Equation (7). Consequently, NAR_4_ has been extended to several tens of millimeters.

Furthermore, if the NAR of distance measurement is expected to be extended, increasing the performing wavelength number of MWI is intuitive. That hypothetical proposal is illustrated in Figure 2 (in gray color), where adding a fifth wavelength to MWI makes NAR_5_ approach 1 m by the mean of the secondary virtual synthesis $\Lambda_{1235}$ (comprised of $\lambda_{1}$, $\lambda_{2}$, $\lambda_{3}$, and $\lambda_{5}$), but $\lambda_{5}$ should also be frequency-locked to the frequency comb at the cost of expense and complexity. Given that NAR_4_ has already reached several tens of millimeters, there are many solutions for the acquisition of course distances with a required precision that is smaller than half of the NAR_4_. In order to make a compact absolute interferometer for real-time and one-shot measurement, it is preferable to adopt direct synthetic wavelength interferometry (DSWI) for the extension of NAR_5_. In DSWI, since two continuous-wavelength lasers with a beat signal of a radio-frequency level pass through the measurement arm together and then through the measuring phase difference of the beat signal between the measurement arm and reference arm, the distance *L* is measured and can be expressed as [28]

(9) $$L=\frac{c}{2\cdot n\cdot f_{\mathrm{DSWI}}}\cdot e_{\mathrm{DSWI}}$$

where $c/f_{\mathrm{DSWI}}$ is regarded as the direct synthetic wavelength of $\lambda_{\mathrm{DSWI}}$, $e_{\mathrm{DSWI}}$ is the detected fractional phase for DSWI, *c* is the light velocity, and *n* is the air refractive index. Apparently, the measurement range of DSWI depends on the beat frequency $f_{\mathrm{DSWI}}$ and has no severe request on the wavelength itself about wavelength stability and position selection, so practically a free-running laser with a wavelength of $\lambda_{5}$ is modulated to generate the direct synthetic wavelength. Moreover, $\lambda_{5}$ can be positioned in the middle area of the comb band far away from the other wavelengths $\lambda_{1}$, $\lambda_{2}$, $\lambda_{3}$ and $\lambda_{4}$, which is handy for next wavelength de-multiplexing. Given the beat frequency at the MHz level and the common precision on phase detection, DSWI readily covers a meter-level range while NAR_4_ and NAR_5_ are bridged, with a high scaling factor resulting from the direct phase detection of synthetic wavelengths instead of the phase synthesis in MWI. To conclude, the advantages of adopting DSWI are undesired for the wavelength calibration of MWI, and that disposal is also suitable for parallel wavelength de-multiplexing and highly efficient for NAR extension.

## 3. Experimental Setup

**Figure 3:**
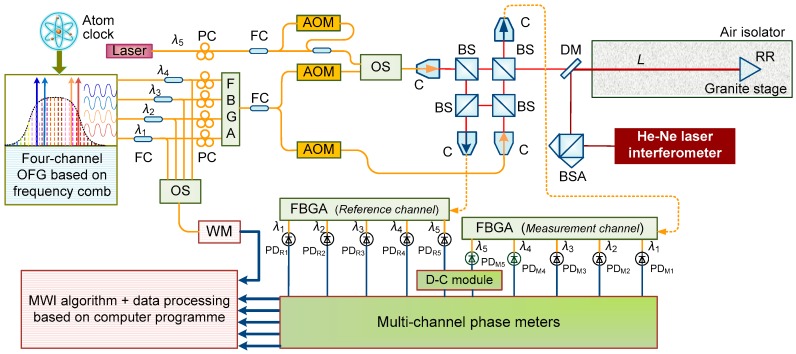
Schematic diagram of the absolute distance measurement (ADM) configuration. PC: polarization controller; FC: fiber coupler; C: collimator; BS: beam splitter; DM: dichroic mirror; BSA: beam splitter assembly; RR: retro-reflector; OS: optical switch; WM: wavelength meter; *L*: targeted distance.

Figure 3 shows the schematic diagram of the ADM interferometer configured in this investigation. Since the frequency comb is crucial as the wavelength ruler to generate four wavelengths for MWI, an Er-doped fiber femtosecond laser, a Menlo Systems C-fiber type, was adopted with its frequency (the repetition rate *f_r_* and carrier-envelop-offset frequency *f_ceo_*) being stabilized to the Rb atomic clock. The frequency of *f_ceo_* was obtained from an *f*-2*f* interferometer [29] and fine-controlled by a phase-locked loop (PLL) to 30 MHz together with the repetition rate to 100 MHz, both traced to the Rb atomic clock. The laser source for MWI here was constructed as an optical frequency generator (OFG) based on the frequency comb [30,31]. A four-channel OFG is comprised of four distributed-feedback (DFB) lasers that are simultaneously phase-locked to the selected modes of frequency comb by PLL control. Before the four separate lasers were fiber-coupled into a single mode fiber through a fiber Bragg grating array (FBGA), a small amount of them was optically switched to a wavelength meter to acquire the accurate wavelengths. The combined laser of the OFG was fiber-coupled into two beams and frequency-shifted by two acoustic-optical modulators (AOMs) with driving signals of 40 MHz and 40.04 MHz. Therefore, such a slight frequency difference resulted in a heterodyne signal of 40 kHz for phase detection. Before the split laser for the measurement arm illuminated the interferometer, an external laser pair generated by mixing two beams, an original beam, and a 40-MHz-frequency-shifted one operated by another AOM were coupled into this arm of the laser source through the optical switch to work as the direct synthetic wavelength laser for DSWI. After traveling through the absolute interferometer comprised of four non-polarization beam splitters, measurement signals and reference signals, including heterodyne signals and synthetic-wavelength beat signals, were collimated to the receiving fibers and then sent to the FBG arrays for wavelength demodulation. The demodulated single wavelengths were detected using highly sensitive photoelectric detectors (PDs) simultaneously. These pairs of detected heterodyne signals for MWI were directly transported to the multi-channel phase meter, while synthetic-wavelength detected signals were frequency-down-converted by the down-conversion (D-C) module for signal processing in advance. The multi-channel phase meter measured the interference phases of five wavelengths simultaneously but individually, and the resulting phases were finally sent to the computer for data processing. For performance evaluation, a 3.0-m-long granite air-bearing stage was built, and high-precision ambient sensors were laid out along the air-bearing stage for compensation of the air refractive index. A commercial He–Ne laser interferometer was installed along the same measurement optical path of the ADM interferometer by a dichroic mirror. In particular, to reduce the influence of temperature variation and intense air flow during long-term demonstration, the granite stage was enclosed by an isolator, which was enwrapped by thick silver papers to maintain homogeneity.

**Figure 4:**
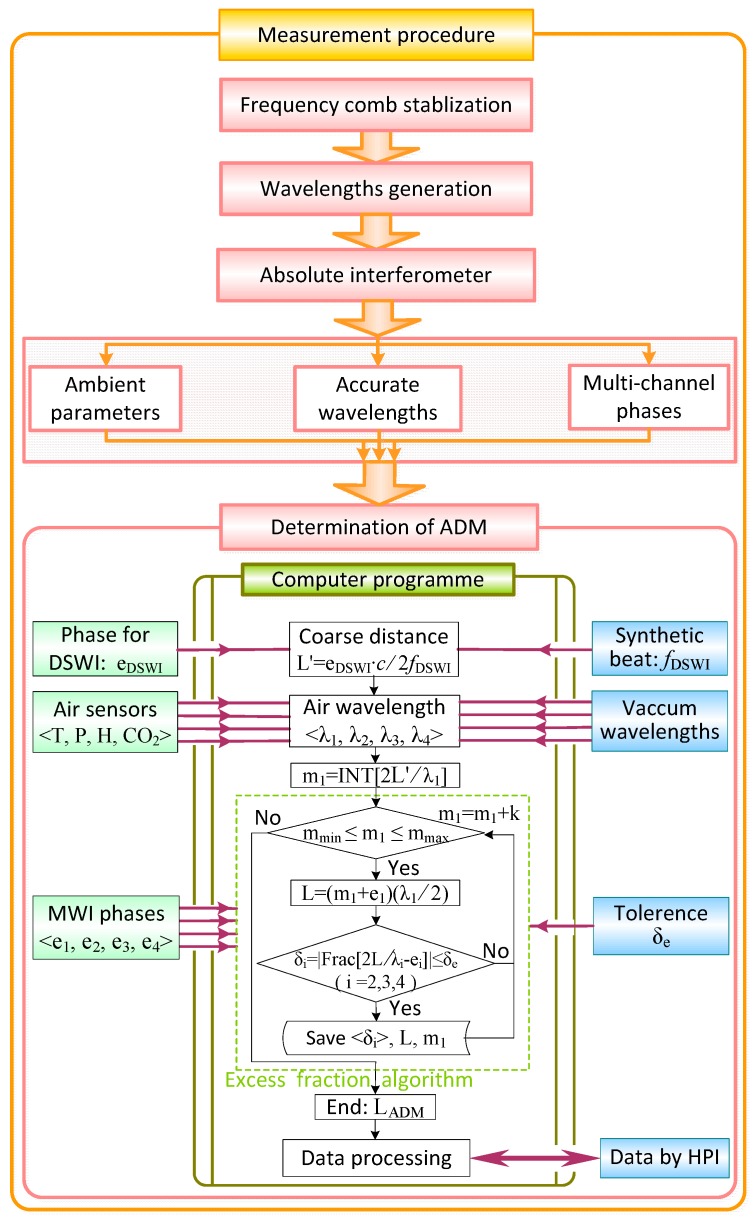
Measuring procedure for real-time and meter-scale absolute distance measurement.

Figure 4 shows the entire measuring procedure. The primary step is the stabilization of the frequency comb, which essentially decides the reliability of ADM by MWI. The second step is wavelength generation, including four-channel continuous wavelengths stabilized for MWI and the synthetic wavelength modulated for DSWI. After illumination of the absolute interferometer, the parameters of wavelengths (settled in the part of wavelengths generation), the multi-channel-detected phases, and the ambient parameters are acquired in parallel. With the acquisition and transmission of the desired parameters, absolute distance is calculated by computer programming based on the excess fraction method. A brief flow chart of the excess fraction algorithm for determining the precise distance is also provided.

## 4. Experimental Results and Discussion

### 4.1. Preparative Test

**Figure 5:**
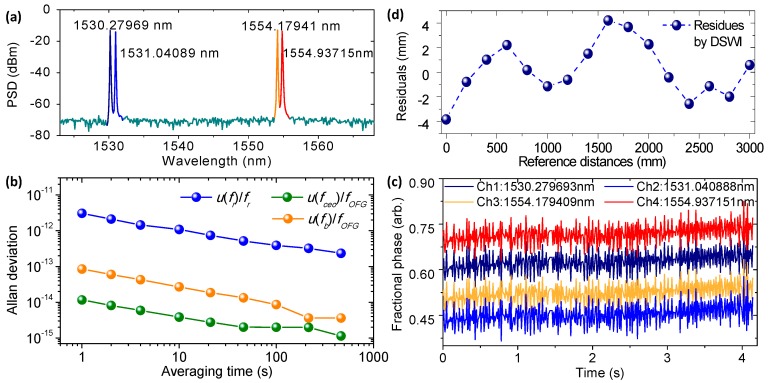
Test results for preparation of real-time and meter-scale absolute distance measurement. (**a**) Parallel generated four wavelengths for MWI. (**b**) Frequency stability evaluation. (**c**) Simultaneously detected phases for MWI in real time. (**d**) Comparative residual distances between DSWI and He–Ne laser interferometer.

The frequency-comb-referenced four wavelengths as OFG of MWI were observed via optical spectrum analyzer, showing a signal-to-noise ratio of more than 50 dB, as is depicted in Figure 5a. The exact wavelengths, sequentially located at 1530.279693 nm, 1531.040888 nm, 1554.179409 nm, and 1554.937151 nm, were obtained by determining the number of comb modes through a high-precision wavelength meter (WS-U 10, High Finesse GmbH, Tübingen, Germany) with an accuracy of 0.2 pm [32]. Such a four-wavelength selection led to a specific NAR chain, depicted in Table 1. On the basis of the exact wavelength position, a scaling factor chain for transition of the neighboring NAR was estimated as $\beta_{2}\approx 59$, $\beta_{3}\approx 38$ and $\beta_{4}\approx 26$, which demands a phase precision better than 0.006 in fraction, according to Equation (7).

Figure 5b shows a frequency stability of frequency-locked terms accomplished in a frequency-comb-referenced OFG. As the frequency of each OFG laser can be expressed as $f_{OFG}=\;N\times f_{r}+f_{ceo}+f_{b}$ in which $f_{r}$, $f_{ceo}$, and $f_{b}$ are the repetition rate of the frequency comb, the carrier-envelope-offset frequency of the frequency comb, and the beat frequency between the N-th comb mode and the frequency-locked laser, respectively [31], the frequency stability of the locked laser can be expressed in a fractional form of $u\left(f_{OFG}\right)/f_{OFG}=\sqrt{{\left(u\left(f_{r}\right)/f_{r}\right)}^{2}+{\left(u\left(f_{ceo}\right)/f_{OFG}\right)}^{2}+{\left(u\left(f_{b}\right)/f_{OFG}\right)}^{2}}$. The PLL-locked signals of $f_{r}$, $f_{ceo}$, and $f_{b}$ were observed via a radio-frequency spectrum with a signal-to-noise ratio of better than 30 dB. The variations of these controlled signals were measured using a frequency counter referenced to the Rb atomic clock with the type of Model FS725-Stanford Research Systems and were estimated in terms of the Allan deviation with the averaging time from 1 to 500 s as shown in Figure 5b. Specifically, the stability of OFG frequency in 10 s averaging time was synthesized as 1.090 × 10^−12^, while those of $f_{r}$, $f_{ceo}$, and $f_{b}$ were recorded as 1.089 × 10^−12^, 3.819 × 10^−15^, and 2.693 × 10^−14^, respectively.

Figure 5c shows the interference phases of the four wavelengths simultaneously detected at the output ports of the multi-channel phase meters, where all the phases are given in terms of decimal fraction with an update rate of 10 ms, and phase precision for each channel is assessed to be better than 0.004 in fraction. Because all the four wavelengths passed the common optical path of the absolute interferometer, all measured phases yielded almost the same variation and reached an excellent coincidence with each other in front of the optical path length change. Such dynamic test results affirm the real-time measurement capability of multi-channel phase meters.

To validate the feasibility of DSWI to further extend the NAR and to smoothly bridge NAR_4_ and NAR_5_, a linear comparison was deliberately carried out between DSWI and the He–Ne laser interferometer (Agilent 5530, HPI, Santa Clara, CA, USA). A free-running DFB laser in the wavelength area of 1540 nm was embedded as the fifth wavelength and was later AOM-frequency-shifted by 40 MHz for DSWI, so the extended range of NAR_5_ was up to 3.75 m with a high scaling factor of near 90. Figure 5d presents the residual distances from the direct reading data during a linear movement of 3.0 m. This result shows an accuracy of 8.0 mm in peak-to-valley and 2.2 mm in standard deviation, confirming the feasibility for further NAR extension based on measurement accuracy of DSWI, which is smaller than half of the NAR_4_ over the traveled range. Moreover, the measurable range of DSWI is readily enlarged to several tens of meters by choosing a low frequency shift of AOM if MWI permits it.

### 4.2. Linear Distance Measurement

**Figure 6:**
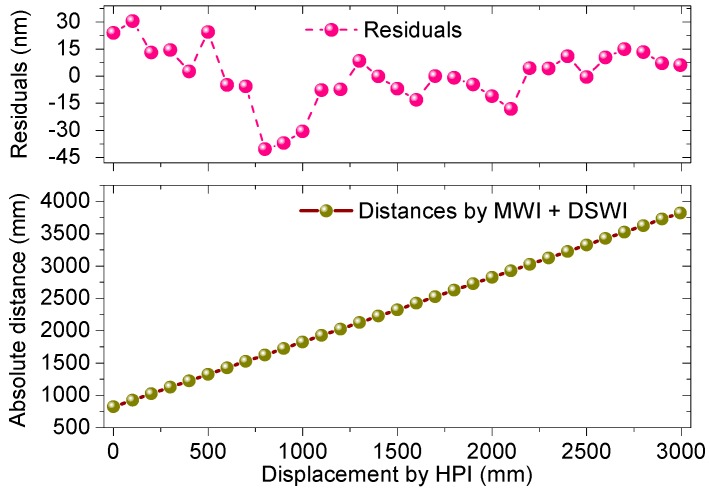
Linear comparison of the ADM interferometer with the He–Ne laser interferometer over a 3.0 m axis distance.

Figure 6 shows a linearity test result that was performed by the integration of MWI and DSWI over a 3.0 m axis distance repeatedly with a 100 mm step. The ADM measurement result was compared with that of the He–Ne interferometer, where the zero datum of the latter was set to an offset distance of 0.8 m to equal the actual distance with the ADM interferometer. Two measurement results, the absolute distance by MWI and the displacement by Agilent 5530 (HPI), show a slight discrepancy with a 70.8 nm residual error in peak-to-valley and 16.4 nm in standard deviation under linear fitting. Given the long measured range, the maximum residual error reaches the level of 1.3 × 10^−8^ in the fractional term, which in fact approaches an inherent uncertainty of the refractive index of air [33]. This linearity test indicates that the ADM result is comparable to the result measured by the He–Ne laser interferometer, which proves a capability of large NAR distance measurement for several meters. A distance determination of longer range demands highly precise phase detection and a more accurate compensation for air refractive index, referring to the MWI retrieval algorithm.

### 4.3. Long-Term Comparison

**Figure 7:**
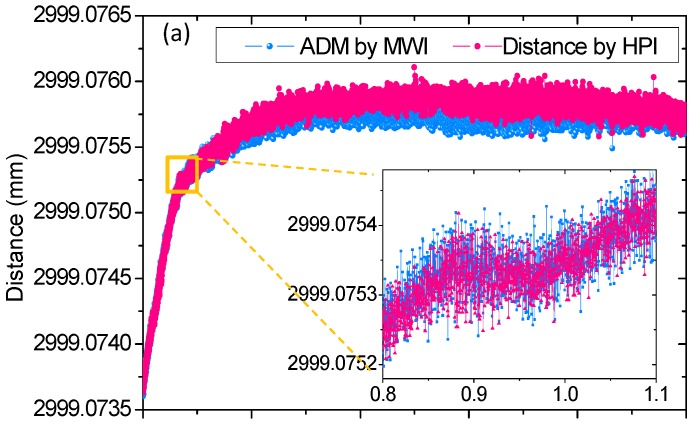

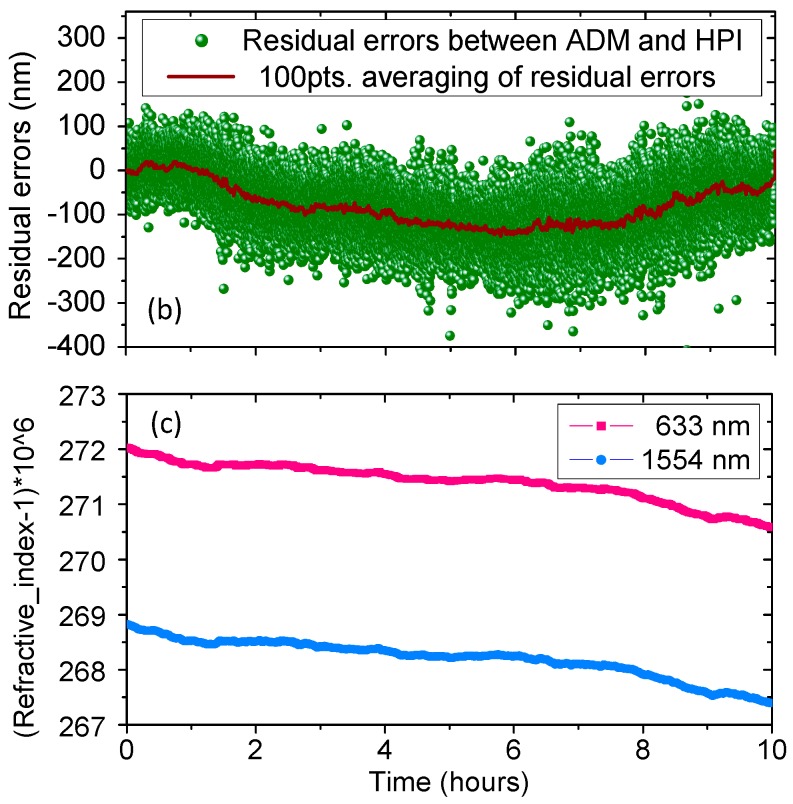
10 h monitored results. (**a**) Time-traces of measured distances by ADM and HPI. (**b**) Distance residues between ADM and HPI readings. (**c**) Air refractive index for ADM and HPI.

To evaluate the long-term measurement stability and capability of the ADM prototype, a 10 h comparison between ADM by MWI+DSWI and HPI was demonstrated, and the analyzed result is shown in Figure 7. During this demonstration, the target mirror was positioned at the far end of the granite guide way and remained stationary at the position of the last measuring point of linear comparison in Figure 6. In addition, the air temperature variation was less than 0.05 °C, illustrating small air disturbance and low heat transfer from the outside to the isolator. Figure 7a shows the 10 h distance variation of the ADM and HPI readings acquired by the division of interferometric phase-dependent optical path difference (OPD) and the refractive index of air at a sampling rate of 10 Hz. For convenience, a distance offset was added to the ADM results to unify the initial reading of the ADM and HPI results. The presented results of ADM and HPI both show a distance increase by ~2.2 μm, and they overlap well along the time traces and slightly deviate in the rear part of the measurement. The inset in Figure 7a, with a zoomed time trace from 0.8 to 1.1 h, present the detailed observation on the entangled results, which show good agreement. The inset results also indicate a short-term distance fluctuation of ~140 nm in the measuring system itself, resulting from mechanical vibration, air inhomogeneity, electronic impulse, and so on. Figure 7b shows distance deviations between the 10 h ADM and HPI readings. These residual distances scatter in the region from −300 to 200 nm, which corresponds to a fractional distance change of more than 1 × 10^−7^, with respect to the total distance, and are attributed to two major factors. One is the asynchronous data sampling and the reading of two distinct measurement systems, and the other is the imperfect compensation for the air refractive index. The former is supposed to be reduced through data smoothing. The residues of 100 point averaging plotted in Figure 7b show a slowly varying discrepancy less than 100 nm in the 10 h comparison, corresponding to a fraction of ~3 × 10^−8^ with respect to the total distance, so the random and fast-varying constituent considered as the main part of the former factor was eliminated to a certain extent. This averaging result well interprets the latter as a slowly varying change in air refractive index. Figure 7c plots the air refractive indexes of ADM and HPI by the Ciddor equation based on the monitoring of the environmental parameters of air temperature, pressure, CO_2_ concentration, and humidity. As these ambient sensors possess uncertainties of 5 mK, 2.5 Pa, 41 ppm, and 1%, respectively, the combination of all of these sensing errors leads to a comprehensive uncertainty in the air refractive index of 3.1 × 10^−8^, which is consistent with what the averaging result in Figure 7b revealed in this long-term demonstration.

## 5. Conclusions

We achieved real-time and meter-scale ADM referenced to the frequency comb. This prototype successfully extends the NAR to the meter level, and was confirmed by a linearity test, where performance was compared with that of the conventional laser interferometer, with a fractional residue less than 1.3 × 10^−8^ over a distance of 3.0 m. The long-term comparison demonstrated for 10 h with fractional deviations of ~3 × 10^−8^ after smoothing shows that the ADM interferometer is definitely competent for long-term measurement. In summary, the reported frequency-comb-referenced absolute interferometer achieves ADM with a meter-scale NAR, high precision, a fast measurement speed, and instant acquisition, making it fully potential for precise positioning and ranging in industrial applications and space missions.

## Figures and Tables

**Table 1:** NAR chain and wavelength selection of the constructed multi-wavelength interferometry (MWI).

NAR Chain	NAR_1_ (*λ*_1_/2)	NAR_2_ (∧_14_/2)	NAR_3_ (∧_12_/2)	NAR_4_ (∧_1234_/2)
*λ*_1_ = 1530.279693 nm	√	√	√	√
*λ*_2_ = 1531.040888 nm			√	√
*λ*_3_ = 1554.179409 nm				√
*λ*_4_ = 1554.937151 nm		√		√
Quantity of NAR	765 nm	45 μm	1.7 mm	45 mm
